# 

**Published:** 1909-04

**Authors:** 


### CONTENTS—APRIL, 1909.—VOL. XXIV, NO. .10

ORIGINAL ARTICLES—

Our Unfortunate Insane. By Dr. G. I. King, Elgin, Texas.... 409

Some Remarks on Lacerations of the Perineum; A Plea for More Thoroughness in the Prevention, Also for the Immediate Repair; The Author's Technique. By Addison L. Lincecum, M. D., Louise, Texas.... 413

Personal Liberty. By G. B. Dorrell, M. D.... 416

EDITORIAL DEPARTMENT—

The White Feather in Evidence.... 419

Our State Organization.... 424

EDITORIALETS.... 429

CORRESPONDENCE.... 430

SELECTED.... 433

ABSTRACTS AND SELECTIONS.... 435

BOOKS AND MAGAZINES.... 444

PUBLISHER'S DEPARTMENT.... 446

